# Left Ventricular Non-compaction and Associated Cardiomyopathy Presenting With Cardiac Failure: A Case Report

**DOI:** 10.7759/cureus.59265

**Published:** 2024-04-29

**Authors:** Waqas Azhar, Gurjot Singh, FNU Saveeta, Didar Singh, Tanya Ratnani, Deepak Singla, Meet Popatbhai Kachhadia, Ninia Goyal, Piyush Puri

**Affiliations:** 1 Internal Medicine, Memorial Medical Center, Springfield, USA; 2 Internal Medicine, Saint John's Hospital, Springfield, USA; 3 Internal Medicine, Southern Illinois University School of Medicine, Springfield, USA; 4 Hospital Medicine, Springfield Clinic, Springfield, USA; 5 Internal Medicine, Maharaj Sawan Singh Charitable Hospital, Beas, IND; 6 Internal Medicine, People's University of Medical and Health Sciences, Nawabshah, PAK; 7 Internal Medicine, Government Medical College, Bilaspur, Bilaspur, IND; 8 Internal Medicine, Adesh Institute of Medical Science and Research, Bathinda, IND; 9 Internal Medicine, Pandit Dindayal Upadhyay (PDU) College, Civil Hospital Campus, Rajkot, IND; 10 Internal Medicine, Chirayu Medical College and Hospital, Bhopal, IND

**Keywords:** alcohol use, cocaine abuse, cardiac manifestations, cardiomyopathy, left ventricular non-compaction cardiomyopathy (lvnc)

## Abstract

The characteristic structural anomaly of the heart in the left ventricular non-compaction (LVNC) is identified with a prominent layer of the trabecular meshwork, thin compacted myocardium, and intertrabecular recesses within the depths of the left ventricle. Despite growing clinical recognition, the prevalence of LVNC in adults and the full clinical spectrum remain poorly explored. The disease shows heterogeneous phenotypes from an asymptomatic presentation to severe cardiac complications like cardiac failure, arrhythmias, and thromboembolic events. Current diagnostic practices for LVNC lack standardized guidelines, making patient management difficult. We here report a case of an adult patient who presented with features of congestive cardiac failure and on detailed imaging with echocardiogram and magnetic resonance imaging (MRI) was diagnosed to have LVNC. We here also emphasize that there is a great need for refined diagnostic criteria that include genetic, clinical, and imaging data. Cases of LVNC with full-blown phenotypic expression should be used for diagnostic criteria.

## Introduction

Left ventricular non-compaction (LVNC) cardiomyopathy is a new and yet unclassified cardiomyopathy with an estimated prevalence of 0.014-0.17% which involves a distinct heart morphology where the left ventricular wall is marked by a prominent trabecular meshwork, thinly compacted myocardium, and substantial intertrabecular recesses, commonly referred to as a spongy myocardium. This condition's impact spans across age groups, with its prevalence in adults still largely unknown. The clinical presentation is notably varied, encompassing asymptomatic cases to severe cardiac dysfunctions. Management primarily addresses symptomatic relief, particularly heart failure, arrhythmias, and sudden cardiac death prevention. We detail a case highlighting the diagnostic journey and management strategy for a patient with LVNC-associated cardiomyopathy and heart failure [1,2].

## Case presentation

A 45-year-old African American woman who visited the outpatient department with a history of hypertension, diabetes mellitus, and non-ischemic cardiomyopathy was admitted with shortness of breath and a persistent cough. She denied any history of fever, chills, sputum production, leg swelling, or difficulty breathing while lying flat or sleeping. The patient had a positive family history of diabetes mellitus and hypertension in her mother. She was on multiple medications, such as clonidine, furosemide, lisinopril, metformin, and potassium chloride, but her compliance was questionable.

Her vital signs included a temperature of 36.8°C, a heart rate of 112 bpm, a blood pressure of 177/115 mmHg, and a respiratory rate of 18 breaths per minute, with an oxygen saturation of 100% on room air. Physical examination noted tachycardia and basal crackles in the lungs. Initial labs showed a white cell count of 6900/mm³, hemoglobin of 11.7 g/dL, and platelets of 334,000/mm³. Serum sodium was 140 millimoles per liter, potassium 3.6 millimoles per liter, chloride 102 millimoles per liter, bicarbonates 26 millimoles per liter, blood urea nitrogen 13 mg per deciliter, and creatinine 0.7 mg per deciliter. Serum troponin was 0.03 nanograms per milliliter, and brain natriuretic peptide was 2408 picograms per milliliter. Serum albumin was 3.3 grams per deciliter, serum bilirubin was 0.7 milligram per deciliter, total protein was 6.2 grams per deciliter, alanine aminotransferase was 96 international units per liter, and aspartate aminotransferase was 115 international units per liter. Cardiomegaly and mild pulmonary vascular congestion were revealed on chest X-ray.

**Figure 1:**
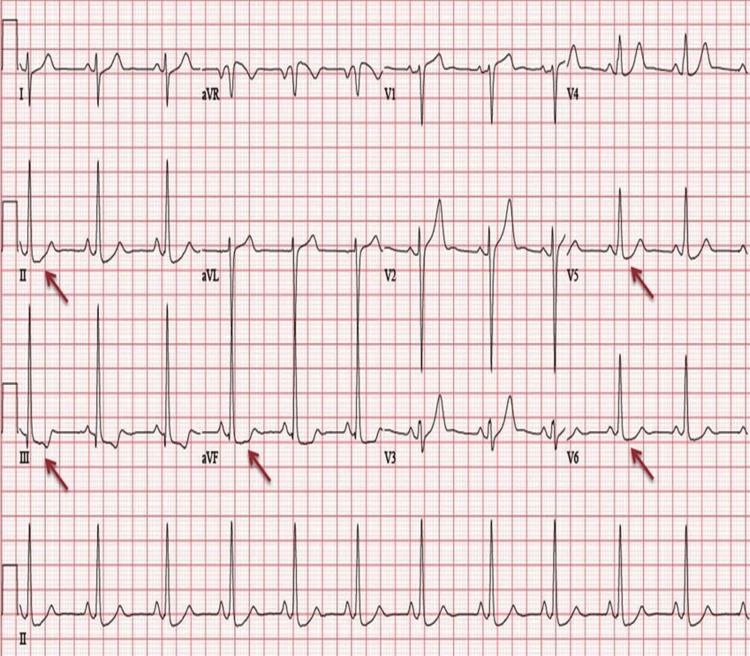
ECG findings showing left ventricular hypertrophy and ST segment depression in inferolateral leads. ECG: electrocardiogram

The cardiology department was consulted, and she was admitted for further management. The patient was started on intravenous diuretics and an antihypertensive regimen. A 12-lead electrocardiogram (ECG) fulfilled the criteria of left ventricular hypertrophy and had a notable ST segment depression in inferolateral leads (Figure 1).

**Figure 2:**
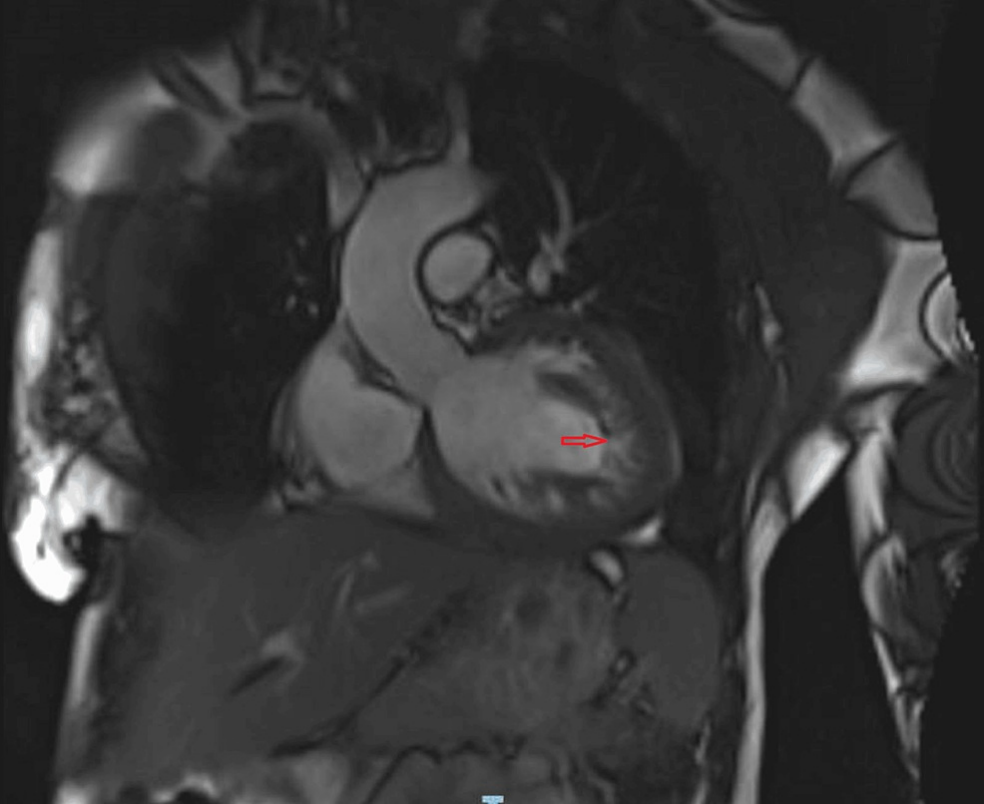
Cardiac MRI showing increased left ventricular thickness. MRI: magnetic resonance imaging

**Figure 3:**
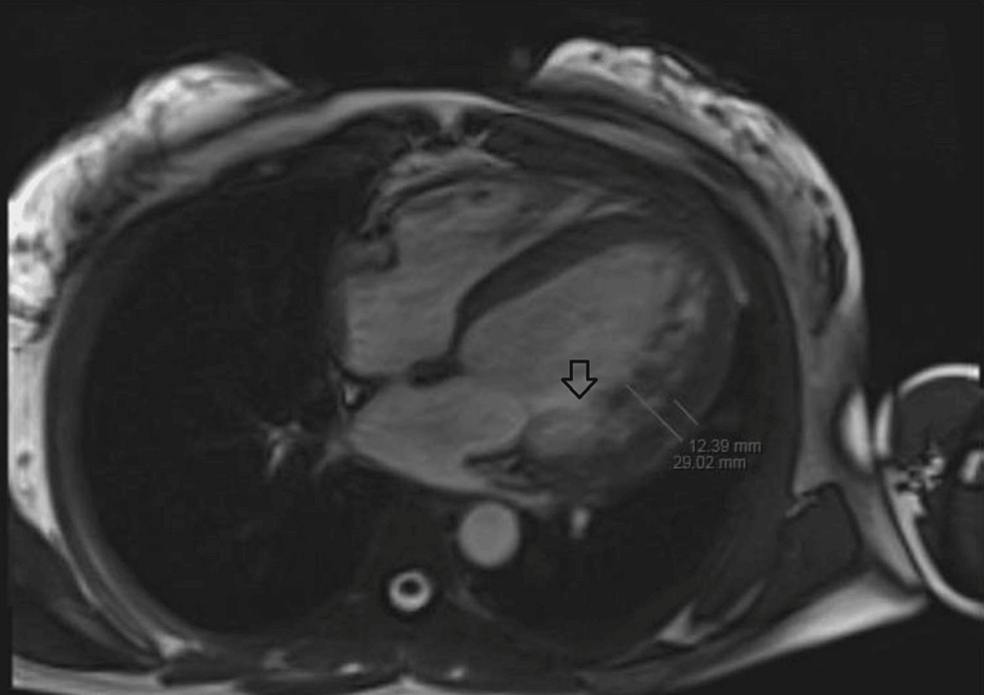
Arrow pointing towards increased left ventricular thickness and trabeculations on cardiac MRI. MRI: magnetic resonance imaging

An echocardiogram revealed a significantly reduced left ventricular ejection fraction of 29%, indicating severe systolic dysfunction, and grade II diastolic dysfunction characterized by multiple wall motion abnormalities. It also showed a moderately increased thickness in the left ventricular wall, with noticeable trabeculations and intertrabecular recesses. Subsequent magnetic resonance imaging (MRI) of the heart confirmed the presence of dilated left ventricular cardiomyopathy, accompanied by global hypokinesia and diminished left ventricular function (Figure 2). Notably, the MRI highlighted an increased thickness in the three most prominent trabecular areas compared to the thin compacted myocardium, with a ratio exceeding 2:1 (Figure 3). This finding is crucial as a maximal end-systolic ratio of non-compacted to compacted layers greater than 2 is the primary diagnostic criterion for LVNC.

Following treatment with intravenous furosemide, which led to adequate diuresis, the patient's symptoms significantly improved. She was subsequently discharged on a regimen of ACE inhibitors, beta-blockers, and continued furosemide to manage her condition effectively.

## Discussion

LVNC is defined as a morphological variation in the ventricle wall, characterized by a pronounced layer of trabecular meshwork, a thin compacted myocardium, and deep intertrabecular recesses connected to the left ventricular cavity. The American Heart Association recognizes it as an independent genetic cardiomyopathy. Meanwhile, the World Heart Federation and the European Society of Cardiology have classified it as an unclassified cardiomyopathy. LVNC affects both pediatric and adult populations. In children, LVNC often coexists with other congenital cardiac anomalies and muscular disorders. Despite advances in imaging techniques that have increased documented cases, the prevalence of LVNC in adults remains unclear [1-3].

The clinical presentation of LVNC varies widely, ranging from asymptomatic cases to severe complications, including heart failure, arrhythmias, systemic thromboembolism, and sudden cardiac death [1]. LVNC can be inherited as an autosomal dominant or X-linked recessive disorder. It is primarily caused by disturbances in molecular signaling due to mutations in genes encoding sarcomere, mitochondrial, cytoskeletal, and ion channel proteins. A large study of the LVNC adult population revealed that 52% of cases were sporadic with no genetic mutation, 32% had a genetic mutation, and only 16% were familial cases without a genetic mutation. Notably, 71% of cases with genetic mutations exhibited mutations in the MYH7, MYBPC3, and TTN genes [2]. Some theories suggest LVNC results from the incomplete maturation of fetal myocardial tissue. However, recent research indicates that the compacted myocardium layer grows intrinsically, not from fetal trabecular tissue, with only papillary muscles developing from such tissues. No one dominant genotype has been linked to excessive trabeculations [1-4].

Currently, there is no consensus or standardized guidelines for diagnosing LVNC. Various criteria have been proposed over the years. Chin et al. suggested measuring the epicardial distance to the trabeculation trough (x) against the epicardial distance to the trabeculation peak (y) in a long-axis diastolic view, diagnosing LVNC if the x/y ratio is less than 0.5 [5]. Jenni et al. recommended a diagnosis based on a maximal end-systolic short-axis trabeculated layer thickness being twice that of the adjacent compacted layer, in the absence of other cardiac anomalies and with color Doppler showing direct blood perfusion from the ventricular cavity into the intertrabecular spaces [6]. Stöllberger et al. advised diagnosing LVNC if more than three trabeculations protrude from the left ventricular wall, apically to the papillary muscles in one imaging plane, combined with perfusion of intertrabecular spaces from the ventricular cavity, later suggesting a combination with Jenni et al.'s criteria for more accuracy [7].

Cardiac MRI offers additional diagnostic support. Petersen et al. recommended a trabeculated-to-compacted layer ratio greater than 2.3, measured in an end-diastolic long-axis view excluding the apex, for an accurate LVNC diagnosis. However, there's a general disagreement in 35% of cases on making a diagnosis, indicating that applying all existing criteria is not practical. The presence of one positive and one negative criterion may lead to confusion [2]. Differentiating LVNC from diastolic/hypertrophic cardiomyopathy is challenging, as thickness ratios between trabeculated and normal myocardium in other conditions do not exceed a 2:1 ratio. Studies have identified incidental LVNC findings in athletes with intact myocardial function, suggesting a multidisciplinary approach could help distinguish between benign and pathological LVNC [2,3,5].

An incidental diagnosis of LVNC typically follows a stable course over the years. However, patients with heart failure symptoms, a history of arrhythmias, or enlarged left atria often experience an unstable clinical course and poor prognosis. The treatment for heart failure in LVNC patients aligns with the guidelines of the American College of Cardiology. Family screening is advised for patients with a diagnosis of heart failure [3-5].

Our patient had multiple risk factors for cardiomyopathy, including cocaine and alcohol abuse, along with uncontrolled hypertension. Nonetheless, the incidental discovery of LVNC may also have contributed to her condition. Familial screening of this patient is likely to benefit her relatives.

## Conclusions

There is very little understanding of the LVNC disease process, associated cardiomyopathy, and other complications. Currently, there is no generalized consensus, guidelines, or diagnostic criteria for LVNC, nor has its natural disease course and prevalence been well-studied. Despite this, LVNC cases are now frequently diagnosed in clinical practice. It is proposed that LVNC cases exhibiting full-blown phenotypic expression and heart failure should be considered true cases of LVNC. These cases should form the basis for developing a better definition and diagnostic criteria, incorporating familial, clinical, and genetic information. Advanced multimodal imaging techniques should also be utilized to better understand the morphology and trabeculation in LVNC patients.

## References

[1] Ichida F (2020). Left ventricular noncompaction - risk stratification and genetic consideration. J Cardiol.

[2] Abela M, D'Silva A (2018). Left ventricular trabeculations in athletes: epiphenomenon or phenotype of disease?. Curr Treat Options Cardiovasc Med.

[3] Arbustini E, Favalli V, Narula N, Serio A, Grasso M (2016). Left ventricular noncompaction: a distinct genetic cardiomyopathy?. J Am Coll Cardiol.

[4] Ikeda U, Minamisawa M, Koyama J (2015). Isolated left ventricular non-compaction cardiomyopathy in adults. J Cardiol.

[5] Chin TK, Perloff JK, Williams RG, Jue K, Mohrmann R (1990). Isolated noncompaction of left ventricular myocardium. A study of eight cases. Circulation.

[6] Jenni R, Oechslin E, Schneider J, Attenhofer Jost C, Kaufmann PA (2001). Echocardiographic and pathoanatomical characteristics of isolated left ventricular non-compaction: a step towards classification as a distinct cardiomyopathy. Heart.

[7] Stöllberger C, Finsterer J, Blazek G (2002). Left ventricular hypertrabeculation/noncompaction and association with additional cardiac abnormalities and neuromuscular disorders. Am J Cardiol.

